# Combination of TrxR1 inhibitor and lenvatinib triggers ROS-dependent cell death in human lung cancer cells

**DOI:** 10.7150/ijbs.86160

**Published:** 2024-01-01

**Authors:** Peisen Zheng, Yiqun Xia, Xin Shen, Hui Lu, Yinghua Chen, Chenxin Xu, Chenyu Qiu, Yafei Zhang, Peng Zou, Ri Cui, Xiaoying Huang

**Affiliations:** 1 Pulmonary Division, Wenzhou Key Laboratory of Interdiscipline and Translational Medicine, The First Affiliated Hospital of Wenzhou Medical University, Wenzhou Medical University, Wenzhou, Zhejiang, China.; 2 Wenzhou Key Laboratory of Heart and Lung, The First Affiliated Hospital of Wenzhou Medical University, Wenzhou Medical University, Wenzhou, Zhejiang, China.; 3 School of Pharmaceutical Sciences, Wenzhou Medical University, Wenzhou, Zhejiang, China.

**Keywords:** TrxR1, Lenvatinib, Lung cancer, Auranofin, ROS

## Abstract

Lung cancer is one of the most lethal diseases in the world. Although there has been significant progress in the treatment of lung cancer, there is still a lack of effective strategies for advanced cases. Lenvatinib, a multi-targeted tyrosine kinase inhibitor, has achieved much attention due to its antitumor properties. Nevertheless, the use of lenvatinib is restricted by the characteristics of poor efficacy and drug resistance. In this study, we assessed the effectiveness of lenvatinib combined with thioredoxin reductase 1 (TrxR1) inhibitors in human lung cancer cells. Our results indicate that the combination therapy involving TrxR1 inhibitors and lenvatinib exhibited significant synergistic antitumor effects in human lung cancer cells. Moreover, siTrxR1 also showed significant synergy with lenvatinib in lung cancer cells. Mechanically, we demonstrated that ROS accumulation significantly contributes to the synergism between lenvatinib and TrxR1 inhibitor auranofin. Furthermore, the combination of lenvatinib and auranofin can activate endoplasmic reticulum stress and JNK signaling pathways to achieve the goal of killing lung cancer cells. Importantly, combination therapy with lenvatinib and auranofin exerted a synergistic antitumor effect *in vivo*. To sum up, the combination therapy involving lenvatinib and auranofin may be a potential strategy for treating lung cancer.

## Introduction

Lung cancer is one of the malignancies with the fastest-growing incidence and mortality rates 1. According to the tumor stage, genetic characteristics and the severity of patients, the common treatment methods for lung cancer mainly include surgery, chemoradiotherapy, immunotherapy and molecular targeted therapy 2. Surgery is the mainstay of lung cancer treatment. However, most patients had developed to an advanced stage when it was diagnosed, which determines that chemotherapy is a crucial strategy for treating lung cancer 3, 4. For this reason, finding more potent drugs or drug combinations for lung cancer are imminent. Vascular endothelial growth factor receptor (VEGFR) is a universally recognized anti-tumor target. VEGFR can combine with VEGF to stimulate the growth and resistance of cancer cells 5, 6. Several VEGFR inhibitors have been developed and are being tested for clinical application, among which lenvatinib, cediranib and nintedanib are extensively studied. However, these VEGFR inhibitors are not effective when used alone, for which they are often utilized in combination with other drugs 7-10.

Reactive oxygen species (ROS) are byproduct of cellular metabolism, which are involved in many cell functions, such as signal transduction, proliferation, and DNA damage in cells 11. Oxidative stress is a serious lesion caused by the imbalance of oxidation and antioxidant system in the body, which is mainly manifested by the rise of ROS 12-14. Cancer cells typically display higher ROS level than normal cells, therefore increasing ROS level in cells may be a good way to combat cancer 15-17. Thioredoxin reductase 1 (TrxR1) is a key enzyme in cells to resist oxidative stress, which can effectively eliminate excess ROS in cells. It has been reported that TrxR1 is highly expressed in many tumors and thought to be related to tumor growth, chemotherapy resistance, and adverse prognosis 18-20. In previous research, we confirmed that TrxR1 inhibitors including auranofin, piperlongumine, and WZ26 can raise the ROS level and induce apoptosis of cancer cells 21-23.

In this study, we investigated whether TrxR1 inhibitors can augment the cytotoxic effects of lenvatinib in lung cancer cells. We corroborated that lenvatinib and auranofin can jointly induce cell death and DNA damage, and these effects depend on the activation of the JNK signaling pathway, which is initiated by an increase in ROS level. Besides, the co-administration of lenvatinib and auranofin substantially triggers ROS-dependent ER stress in lung cancer cells. The *in vivo* experiments also showed that lenvatinib and auranofin can synergistically inhibit tumor growth. Overall, our results indicate that the combination of lenvatinib and auranofin has the potential to be a promising treatment option for human lung cancer.

## Materials and methods

### Chemicals and Antibodies

Lenvatinib (LEN) and N-acetyl-L-cysteine (NAC) were acquired from Aladdin Industrial Corporation (Shanghai, China). Auranofin (AF) was gained from TargetMol (Boston, USA). Antibodies including JNK, ATF4, p-JNK were attained from Cell Signaling Technology (Danvers, USA). Antibodies against GAPDH and CHOP were gained from Proteintech Group (Wuhan, China). Antibody of 53BP1 was provided by Novus Biologicals (Littleton, CO, USA).

### Cell culture

Human lung cancer cell lines H1299, H520 and A549 were provided by the Cell Bank of the Chinese Academy of Sciences. These cells were routinely fostered in RPMI 1640 medium involving 10% heat-inactivated FBS. The cells were propagated in a moist incubator accompanied with an atmosphere of 5% CO_2_ at 37℃.

### Cell viability

Cells were fostered in the 6-well plates about 24 hours in an incubator. After that the cells were handled by the designated concentration of lenvatinib or auranofin for 24 hours followed by using trypan blue exclusion to estimate the cell viability. CompuSyn software was used to count combination index (CI) values 24. A CI value less than 1 suggests a synergistic effect, where the combined agent has a greater effect than each individual agent.

### Western blot analysis

Cell or tumor lysates were mixed with 5× loading buffer and then heated at 100°C for 10 min. The same amounts of proteins from each sample were separated by SDS-PAGE at 80 V for 2 hours, followed by transfer to polyvinylidene fluoride membranes and then blocked with 5% non-fat milk (dissolved with TBST) at room temperature for 1.5 hours. After being rinsed with TBST for three times, the membranes were probed with the designated primary antibodies over 6 h at 4°C and then incubated with the suitable secondary antibody at 37°C for 2 hours. Ultimately, the immunoblots were imaged in an image lab system with ECL substrate.

### Detection of ROS

Cells were fostered in the 6-well plates about 24 hours and then handled by LEN or AF alone or the combination of them for designated times. After that, the cells were incubated in RPMI 1640 medium including DCFH-DA probe for 30 min. The ROS content was detected by a fluorescent microscope.

### Immunofluorescence staining

The cells were propagated on bacteria-free round cover glasses and then fostered in the 6-well plates for 24 hours. After that, the cells were handled by LEN or AF alone or the combination of them for 18 hours. The cover glasses were fixed with 4% paraformaldehyde for 15 min and rinsed with PBS three times followed by being blocked by 5% BSA for 1 hour. The 53BP1 antibody was diluted 800 times and then used to probe the cells at 4°C overnight. After that, the cells were rinsed with PBS four times and then probed with the secondary antibody followed by using a Leica fluorescence microscope to obtain the images.

### Xenografts

All athymic BALB/c nude mice were handled according to the Wenzhou Medical University's Policy on the Care and Use of Laboratory Animals. H1299 cells were selected for research, the cells were re-suspended and injected under the skin of mice. When the tumor volume reached almost 100 mm^3^, the mice were randomly divided into four groups with the following treatments: (1) vehicle group, (2) LEN (20 mg/kg) only, (3) AF (3 mg/kg) only, (4) LEN (20 mg/kg) +AF (3 mg/kg). All mice were intragastrically administrated every other day. The tumor volume was surveyed by vernier caliper on the same day before administration and the dynamic change diagram of tumor was made to track its growth. At last, euthanize the mice and carefully remove the tumors, collecting them for further experiments.

### Lentivirus transfection

H520 cells were fostered in the 6-well plates almost 24 hours and then were infected by lentivirus (GeneChem, Shanghai, China) for 12 hours according to the scheme of the reagent manufacturer. After that, the medium containing virus was replaced with fresh medium. The cells were expanded and fostered in the medium containing puromycin.

### siRNA transfection

The cells were seeded into six well plates and cultured for 24 hours, then the siRNA was combined and transfected into the cells using lipo8000 (Beyotime, China), and the detection or subsequent experiments were carried out 72 hours later. The siRNA sequences used were as follows: siTrxR1-1: 5'-CUU UGC AGC UGC GCU CAA ATT-3', siTrxR1-2: 5'-GCA AGA CUC UCG AAA UUA UTT-3', siJNK1-1: 5'-GCU AGU UCU UAU GAA AUG UTT-3', siJNK1-2: 5'-GCU GGU AAU AGA UGC AUC UTT-3', siJNK2-1: 5'-GAU GCU UUG UGG UAU UAA ATT-3', siJNK2-2: 5'-CCC ACC ACC UCA AAU UUA UTT-3'.

### Immunohistochemistry

After being steeped in 4% paraformaldehyde at 4°C for 1 week, the tumor tissues were imbedded in paraffin. Paraffin-imbedded tissues were cut into sections and a microwave was used to perform the antigen retrieval. After using 3% H_2_O_2_ to block endogenous peroxidase activity, the tissue sections were probed with designated primary antibody for 1 hour followed by being incubated with the secondary antibody for 40 min. The tissue sections were dyed by DAB for 5 min. The images were taken by a Leica microscope.

### Statistical Analysis

GraphPad Prism 5.0 software was used to process date. Student's t-test analysis was employed to identify if there are significant differences between the data of different groups. Data was considered statistically significant when the P-value was less than 0.05.

## Results

### TrxR1 is a potential target for human lung cancer

**Figure 1:**
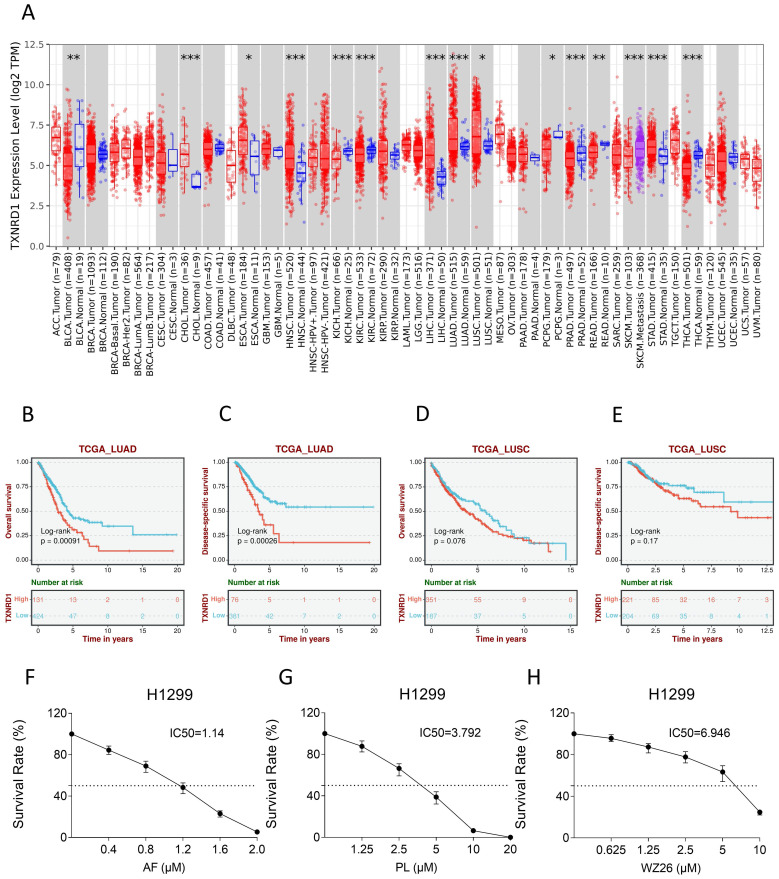
**TrxR1 is a potential target for human lung cancer.** (A) TIMER2.0 database was used to compare variation in TrxR1 expression level between various tumor and normal tissues. (B-E) The Biomarker Exploration of Solid Tumors database was employed to analyze the correlation between TrxR1 expression and the survival of LUAD and LUSC patients. (F-H) Viability of H1299 cells treated with different concentrations of auranofin, piperlongumine or WZ26 for 24 h was measured.

Some studies have shown that TrxR1 inhibitors have good antitumor effects 19, 25, 26. To verify whether TrxR1 is a potential anti-tumor target for lung cancer, we used TIMER2.0 database (http://timer.cistrome.org/) to compare the difference of TrxR1 expression between different tumor tissues and normal tissues. The result showed that the expression of TrxR1 was significantly increased in LUAD and LUSC tissues, and it is more evident in LUAD (Figure 1A). After that, the Biomarker Exploration of Solid Tumors database (https://rookieutopia.com/app_direct/BEST/) was employed to analyze the correlation between TrxR1 expression and the survival of lung cancer patients. The analysis revealed that high expression of TrxR1 is associated with a poor prognosis in lung cancer patients, and this association is more pronounced in LUAD patients (Figure 1B-E). To verify these results, we selected several TrxR1 inhibitors to investigate their toxicity to lung cancer cells. It can be obviously observed that auronofin, piperlongumine, and WZ26 can inhibit the growth of H1299 cells in a concentration dependent manner (Figure 1F-H). These studies all indicate that TrxR1 is a potential target for treating lung cancer.

### TrxR1 inhibitors have synergistic effects with VEGFR or EGFR inhibitors

**Figure 2:**
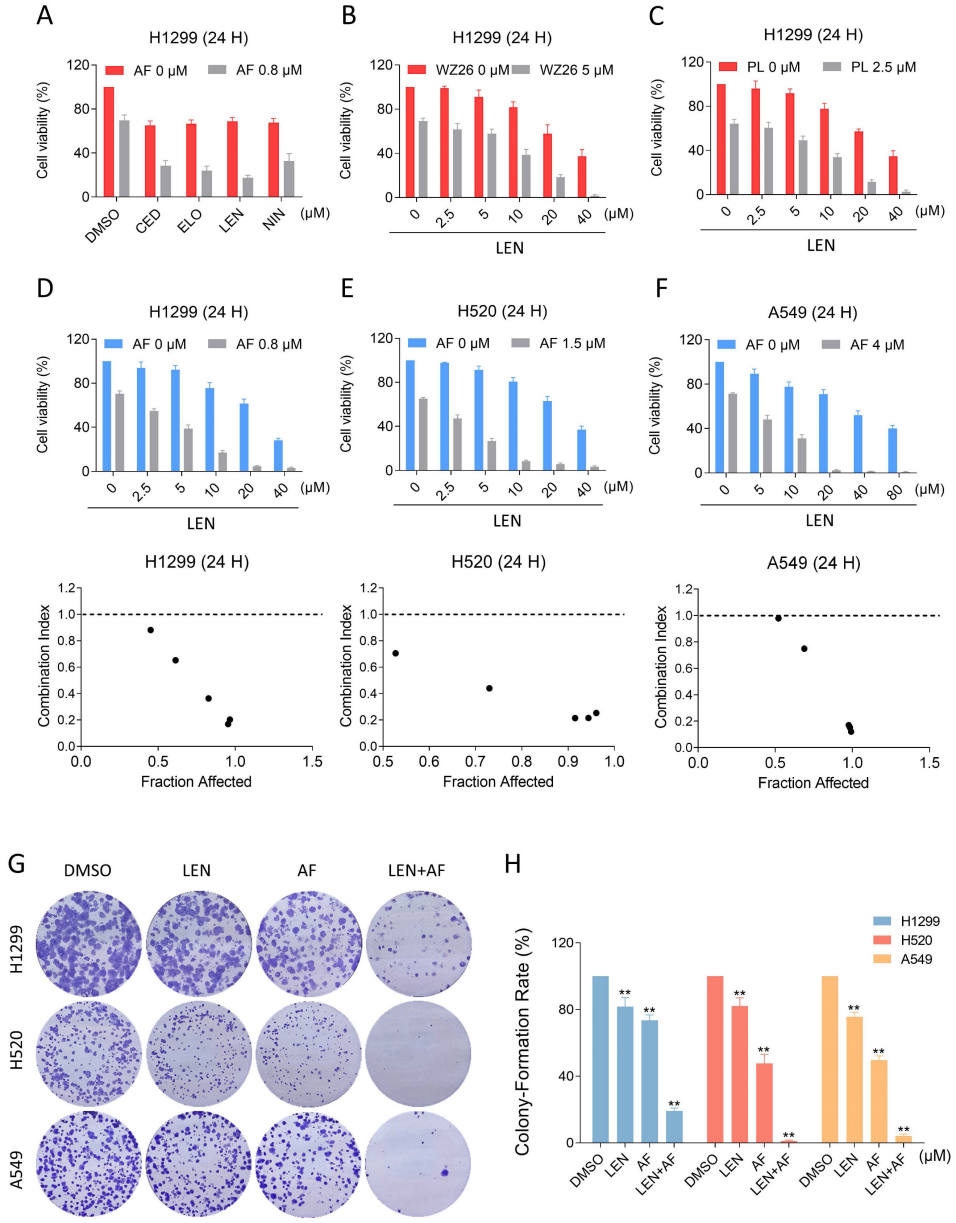
**The combination of TrxR1 inhibitor and VEGFR inhibitor or EGFR inhibitor is toxic to lung cancer cells.** (A) Viability of H1299 cells treated by lenvatinib, cediranib, nintedanib or erlotinib with/without auranofin was measured. (B) Viability of H1299 cells treated by different concentrations of lenvatinib with/without WZ26 was measured. (C) Viability of H1299 cells treated by different concentrations of lenvatinib with/without piperlongumine was measured. (D-F) Viability of H1299, H520 or A549 cells treated by different concentrations of lenvatinib with/without auranofin were measured. CI values were calculated. (G-H) The number of colonies of H1299 (LEN 10 μM, AF 0.2 μM), H520 (LEN 10 μM, AF 0.6 μM) or A549 (LEN 20 μM, AF 3 μM) cells treated by lenvatinib and auranofin alone or the combination of them was measured (** p < 0.01).

In order to explore possible chemotherapy options that include TrxR1 inhibitors, we respectively combined AF with lenvatinib, cediranib, nintdanib, and erlotinib, by which way we found that AF can enhance the cytotoxicity of VEGFR and EGFR inhibitors, especially the lenvatinib (Figure 2A). To verify this result, we combined WZ26 or piperlongumine with lenvatinib and observed significant synergistic effects with both combinations (Figure 2B-C). Subsequently, we selected LEN and AF for further research. We tested the effect of the combination of LEN and AF in H1299, H520 and A549 cells, which all showed significant synergistic effects (Figure 2D-F). To confirm whether this combination can achieve the same synergistic effect in liver cancer, we employed trypan blue exclusion to observe the effects of lenvatinib and auranofin on liver cancer cells. The CI value shows a significant synergistic effect between lenvatinib and auranofin on liver cancer at multiple concentrations (Figure S1A-B). The result of cell cloning experiment also showed that LEN and AF can synergically reduce the number of cell clones of lung cancer (Figure 2G-H).

### The ROS accumulation contributes to synergy of LEN and AF

**Figure 3:**
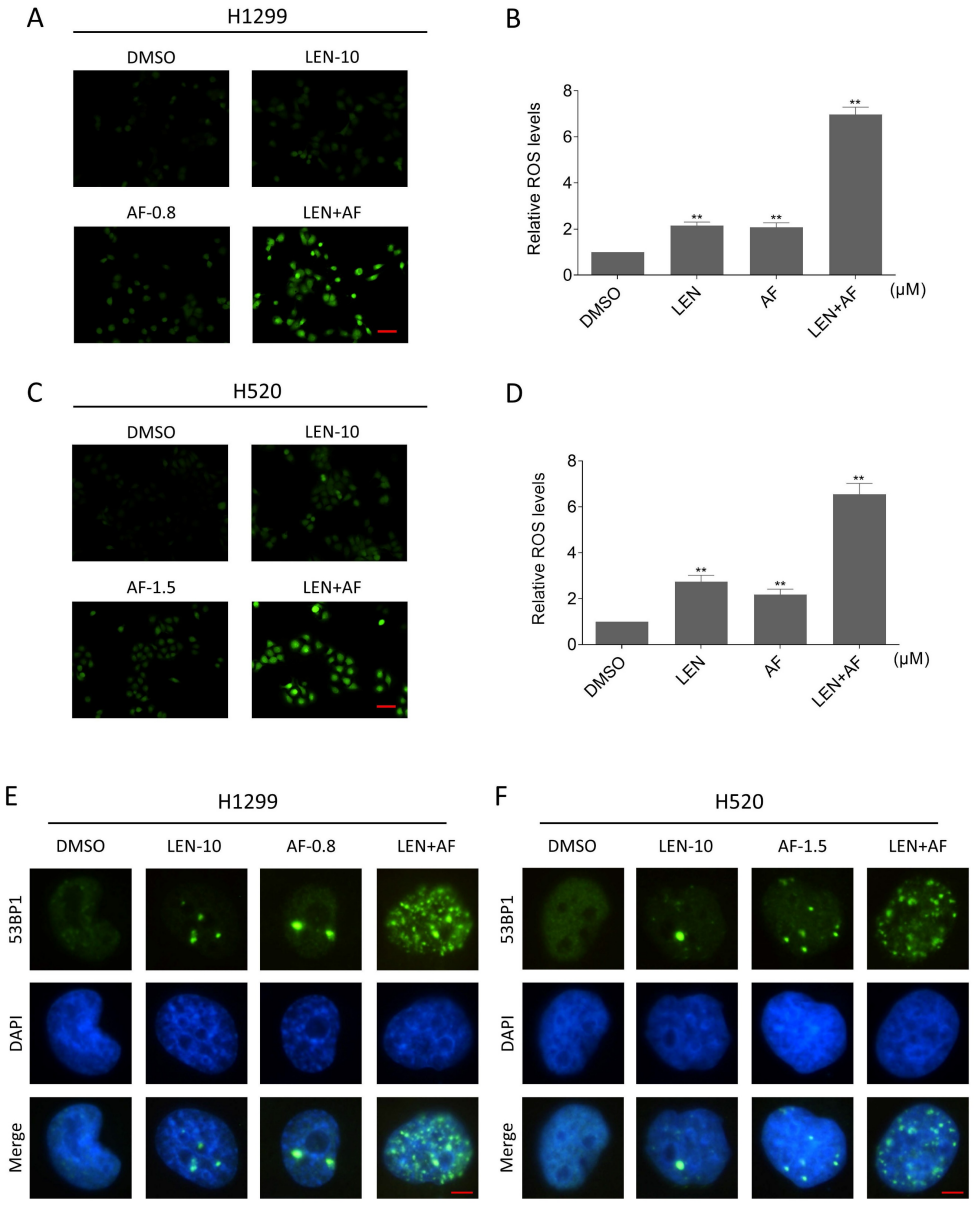
**Lenvatinib and auranofin can synergistically upregulate ROS and induce DNA damage in lung cancer cells.** (A-B) Intracellular ROS level in H1299 cells treated by lenvatinib and auranofin alone or the combination of them was measured. (C-D) Intracellular ROS level in H520 cells treated by lenvatinib and auranofin alone or the combination of them was measured (** p < 0.01). Scale bar = 75 µm. (E-F) Nuclear aggregation of 53BP1 was detected in H1299 and H520 cells treated by lenvatinib and auranofin alone or the combination of them. Scale bar = 5 µm.

**Figure 4:**
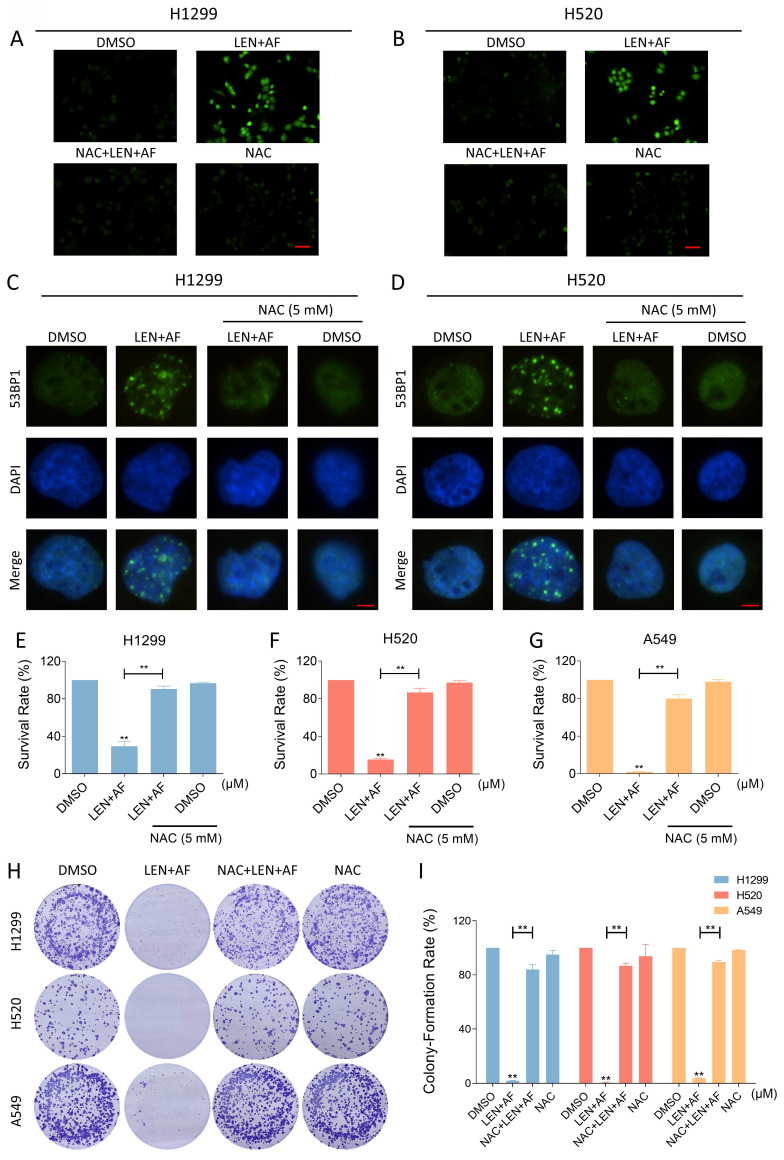
** Eliminating ROS can reverse the synergistic effect of the combination of two drugs.** (A-B) Intracellular ROS level in H1299 (LEN 10 μM; AF 0.8 μM) and H520 (LEN 10 μM; AF 1.5 μM) cells which were pre-treated by NAC was measured after being treated by the combination of lenvatinib and auranofin. Scale bar = 75 µm. (C-D) Nuclear aggregation of 53BP1 was detected in H1299 (LEN 10 μM; AF 0.8 μM) and H520 (LEN 10 μM; AF 1.5 μM) cells which were pre-treated by NAC and then treated by the combination of lenvatinib and auranofin. Scale bar = 5 µm. (E-G) Viability was measured in H1299 (LEN 10 μM, AF 0.8 μM), H520 (LEN 10 μM, AF 1.5 μM) or A549 (LEN 20 μM, AF 4 μM) cells which were pre-treated by NAC and then treated by the combination of lenvatinib and auranofin (** p < 0.01). (H-I) The number of colonies of H1299 (LEN 10 μM, AF 0.2 μM), H520 (LEN 10 μM, AF 0.6 μM) or A549 (LEN 20 μM, AF 3 μM) cells which were pre-treated by NAC was measured after being treated by the combination of lenvatinib and auranofin (** p < 0.01).

TrxR1 is an important enzyme for cellular redox function in protecting against oxidative stress. TrxR1 inhibitor has been reported to upregulate ROS in cancer cells 27-29. In order to explore the specific mechanism underlying the synergy between LEN and AF, we tested the level of ROS in lung cancer. Our findings indicate that the combination of LEN and AF prominently raised the ROS level in H1299 and H520 cells compared to using LEN or AF alone (Figure 3A-D). Besides, through the immunofluorescence assay, the nuclear 53BP1 foci was found prominently increased and accumulated after being handled by the combination of LEN and AF (Figure 3E-F). To explore whether the raised ROS level was related to the cell death caused by the combination of LEN and AF, H1299 and H520 cells were pre-treated by NAC for 1 hour. We found that NAC can eliminate the ROS in cells (Figure 4A-B). Moreover, the increased amount of nuclear 53BP1 foci was reversed by NAC pretreatment (Figure 4C-D). The result of cell viability assay showed that NAC can rescue the cell death caused by the combination of LEN and AF (Figure 4E-G). To verify this conclusion, we conducted a cell cloning experiment and observed that the reduction in the number of cell clones caused by combined treatment was reversed by NAC (Figure 4H-I).

### The ROS-dependent JNK signaling pathway is related to the cell death caused by LEN and AF

**Figure 5:**
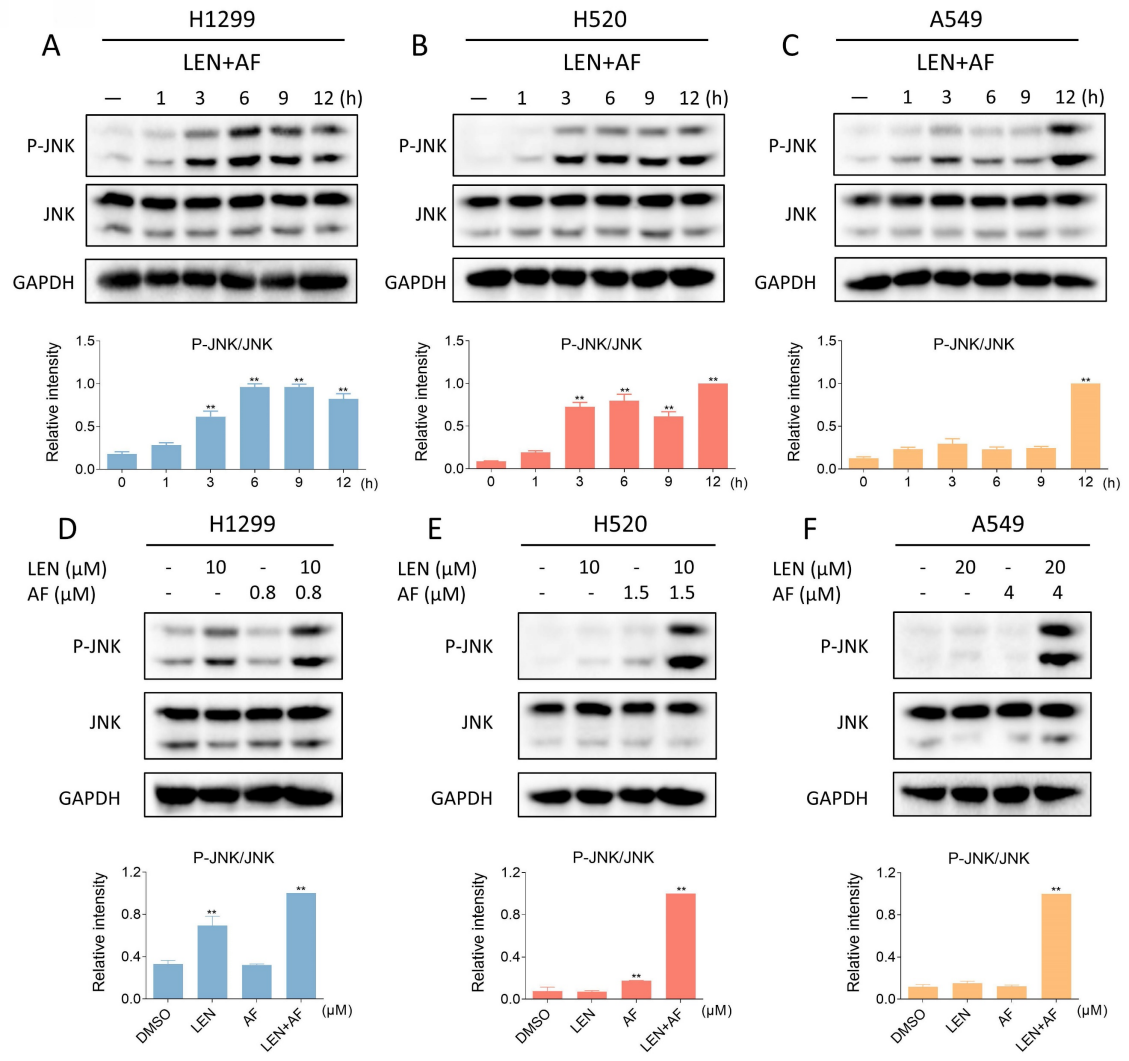
** Lenvatinib and auranofin can synergistically upregulate the phosphorylation of JNK.** (A-C) The level of p-JNK and JNK in H1299 (LEN 10 μM, AF 0.8 μM), H520 (LEN 10 μM, AF 1.5 μM) or A549 (LEN 20 μM, AF 4 μM) cells was detected after being treated by the combination of lenvatinib and auranofin for different durations (** p < 0.01). (D-F) After being treated by lenvatinib and auranofin alone or the combination of them, the level of p-JNK and JNK in H1299, H520 or A549 cells was measured (** p < 0.01).

**Figure 6:**
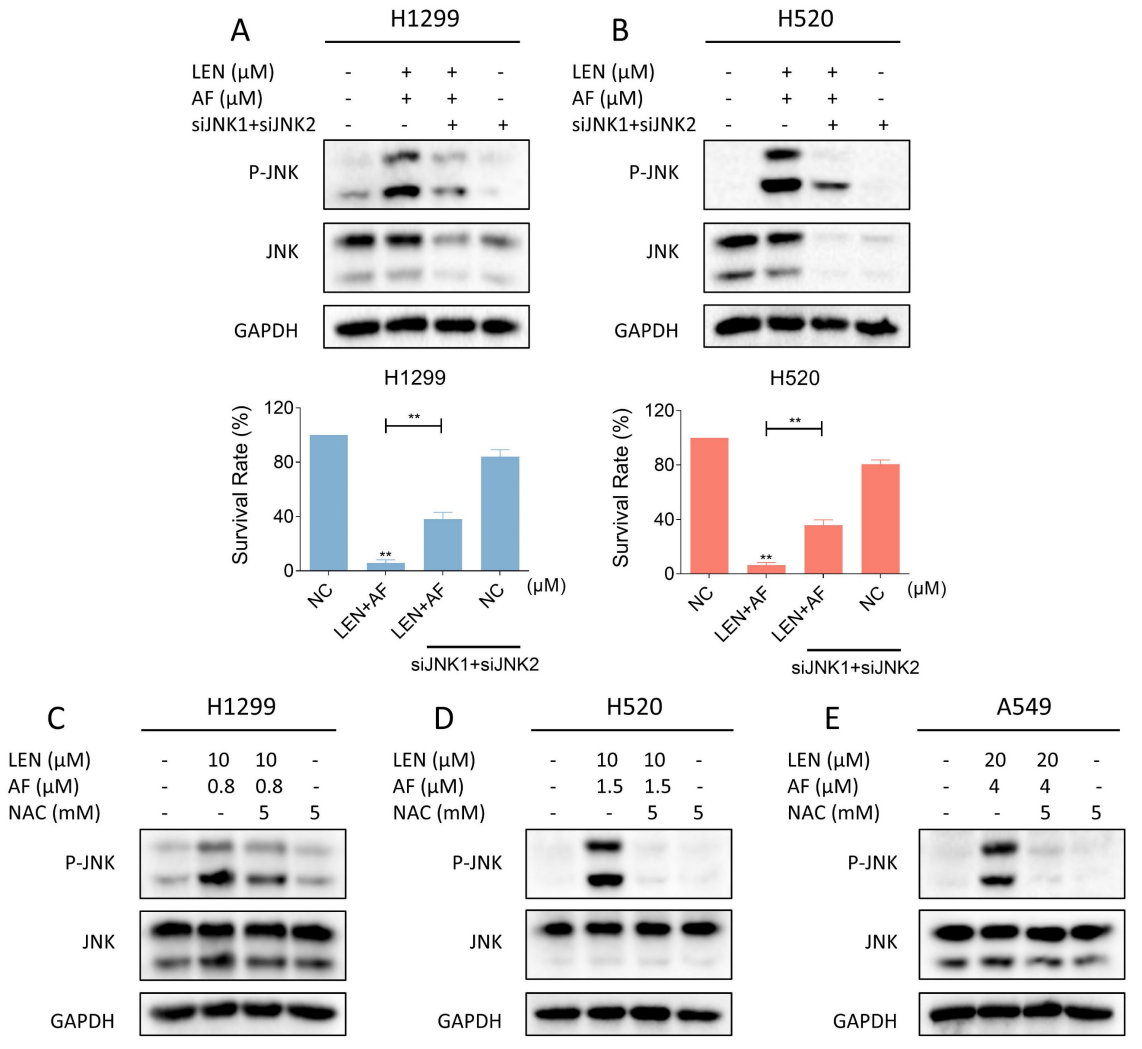
** Reversal of phosphorylated JNK can rescue cell death induced by combination therapy.** (A-B) The level of p-JNK and JNK in H1299 (LEN 10 μM, AF 0.8 μM) and H520 (LEN 10 μM, AF 1.5 μM) cells was detected after being treated by the combination of lenvatinib and auranofin with or without siJNK1 and siJNK2. The viability was also measured (** p < 0.01). (C-E) Cells were treated by NAC for 1 hour before the combination treatment, western blot analysis was then employed to evaluate the level of p-JNK and JNK in H1299, H520 or A549 cells.

Recent findings indicate that the JNK signaling pathway can be triggered by various stimuli, among which oxidative stress is a noteworthy activator 30-32. We then explored the relationship between the JNK signaling pathway and the synergy of LEN and AF. Western blot results showed that the phosphorylation of JNK in H1299, H520 and A549 cells was prominently activated after being treated with LEN combined with AF for different durations (Figure 5A-C). Besides, compared with using LEN or AF alone, LEN combined with AF more significantly activated the JNK signaling pathway (Figure 5D-F). To explore whether the activated JNK signaling pathway is essential for cell death caused by LEN combined with AF, we silenced JNK1 and JNK2 in H1299 and H520 cells using siRNA. We found that the phosphorylation of JNK activated by the combined treatment was blocked by siRNA, and the cell death was also reversed (Figure 6A-B and Figure S2A-B). Further research showed that pretreatment with NAC can reverse the increased phosphorylation of JNK (Figure 6C-E and Figure S2C-E). These findings show that the ROS-dependent JNK signaling pathway is crucial for the combined therapy.

### LEN synergized with AF to induce ER stress in lung cancer

**Figure 7:**
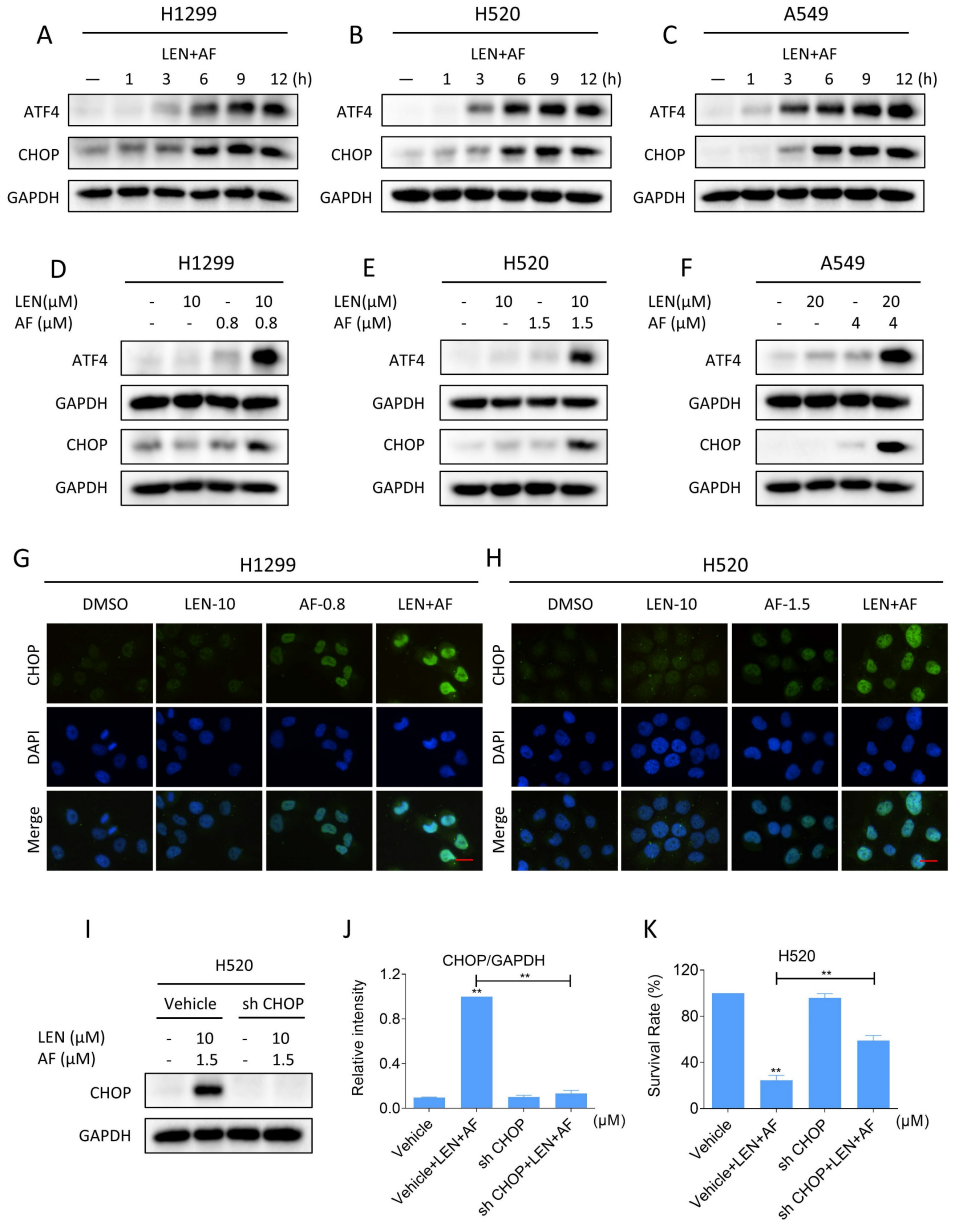
**Lenvatinib and auranofin can synergistically induce ER stress.** (A-C) The level of ATF4 and CHOP in H1299 (LEN 10 μM, AF 0.8 μM), H520 (LEN 10 μM, AF 1.5 μM) or A549 (LEN 20 μM, AF 4 μM) cells was measured after being treated by the combination of lenvatinib and auranofin for different durations. (D-F) The level of ATF4 and CHOP in H1299, H520 or A549 cells was measured after being treated by lenvatinib and auranofin alone or the combination of them. (G-H) The images taken by fluorescence microscopy showed the level of CHOP in H1299 and H520 cells after being treated by lenvatinib and auranofin alone or the combination of them. Scale bar = 25 µm. (I-J) H520 cells will be infected with lentivirus, and the level of CHOP was detected after being treated by the combination of lenvatinib and auranofin for 12 hours (** p < 0.01). (K) H520 cells will be infected with lentivirus, and the viability was measured after being treated by the combination of lenvatinib and auranofin for 24 hours (** p < 0.01).

Elevated ROS level has been proved to be interrelated to the activation of ER stress 33-35. Therefore, we investigated whether LEN and AF can synergistically trigger ER stress. Western blot results showed that combination of LEN and AF can elevate the expression of ATF4 and CHOP (Figure 7A-C and Figure S3A-C). Significantly, compared with using LEN or AF alone, LEN combined with AF more prominently elevated the expression of ATF4 and CHOP (Figure 7D-F and Figure S3D-F). Through the immunofluorescence assay, we further discovered that LEN synergized with AF to promote CHOP aggregation in the nucleus (Figure 7G-H). To validate whether CHOP is essential for the synergy of LEN and AF, we use a lentivirus system to knock down the expression of CHOP (Figure 7I-J). Significantly, the cell death caused by the combination of LEN and AF was rescued by the knockdown of CHOP expression, which indicate that CHOP is essential for the synergy of LEN and AF (Figure 7K).

**Figure 8:**
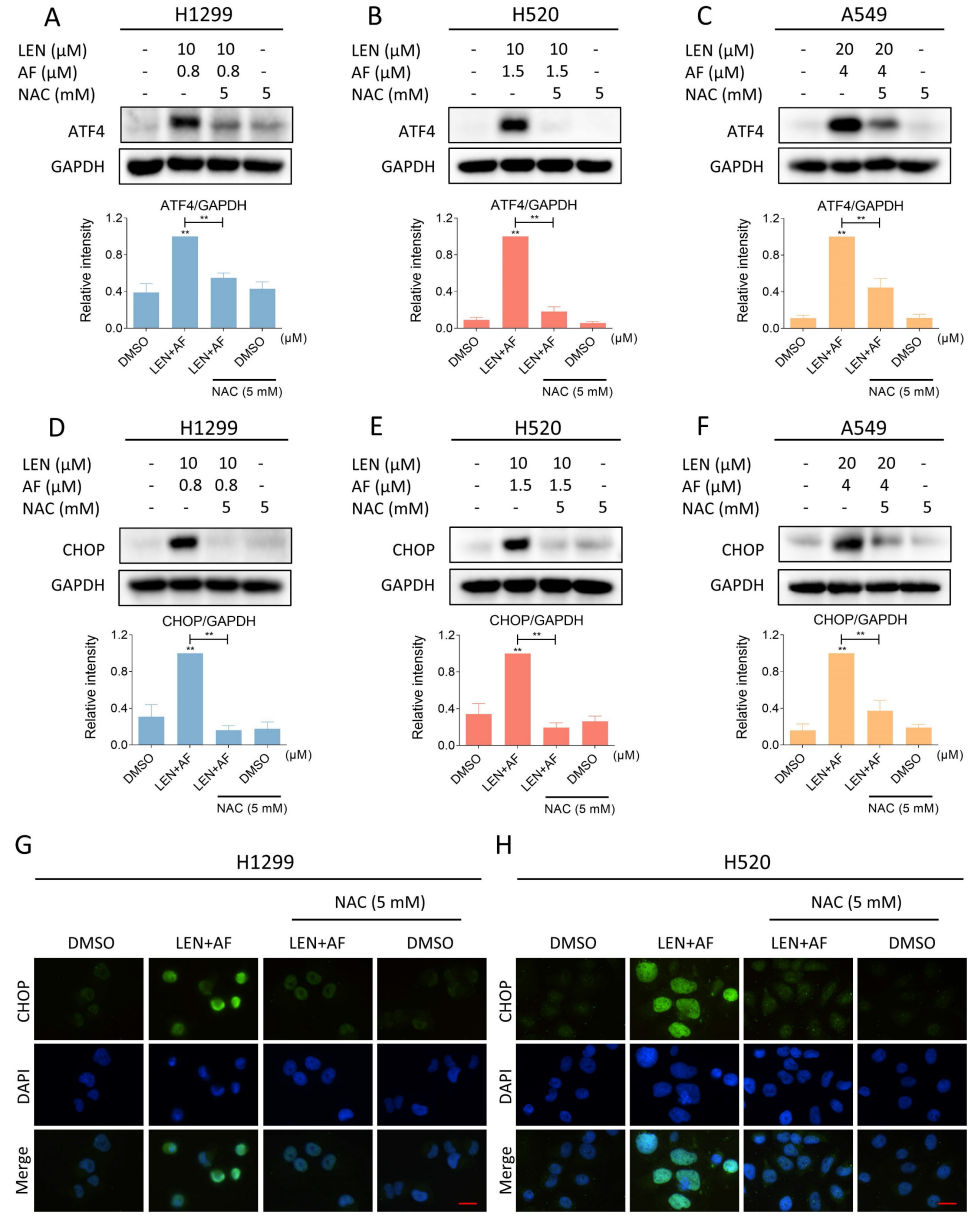
** Eliminating ROS can reverse the activated ER stress.** (A-C) The level of ATF4 in H1299 (LEN 10 μM; AF 0.8 μM), H520 (LEN 10 μM; AF 1.5 μM) and A549 (LEN 20 μM, AF 4 μM) cells which were pre-treated by NAC was measured after being treated by the combination of lenvatinib and auranofin (** p < 0.01). (D-F) The level of CHOP in H1299 (LEN 10 μM; AF 0.8 μM), H520 (LEN 10 μM; AF 1.5 μM) and A549 (LEN 20 μM, AF 4 μM) cells which were pre-treated by NAC was measured after being treated by the combination of lenvatinib and auranofin (** p < 0.01). (G-H) Cells were pre-treated by NAC for 1 hour and then treated by the combination of lenvatinib and auranofin, the images were taken by fluorescence microscopy. Scale bar = 25 µm.

Next, we investigated whether there is an association between the accumulation of ROS and ER stress both evoked by the combination treatment. We pre-treated the cells with NAC for 1 hour and found that the expression of ATF4 and CHOP in H1299, H520 and A549 cells was reversed following the scavenging of ROS (Figure 8A-F). Besides, the immunofluorescence assay also indicated that the CHOP aggregation in nucleus was reversed by the pretreatment of NAC (Figure 8G-H). These results all confirmed that LEN and AF can synergistically upregulate ROS to induce ER stress.

### LEN and siTrxR1 can jointly induce ROS-dependent ER stress and JNK phosphorylation

**Figure 9:**
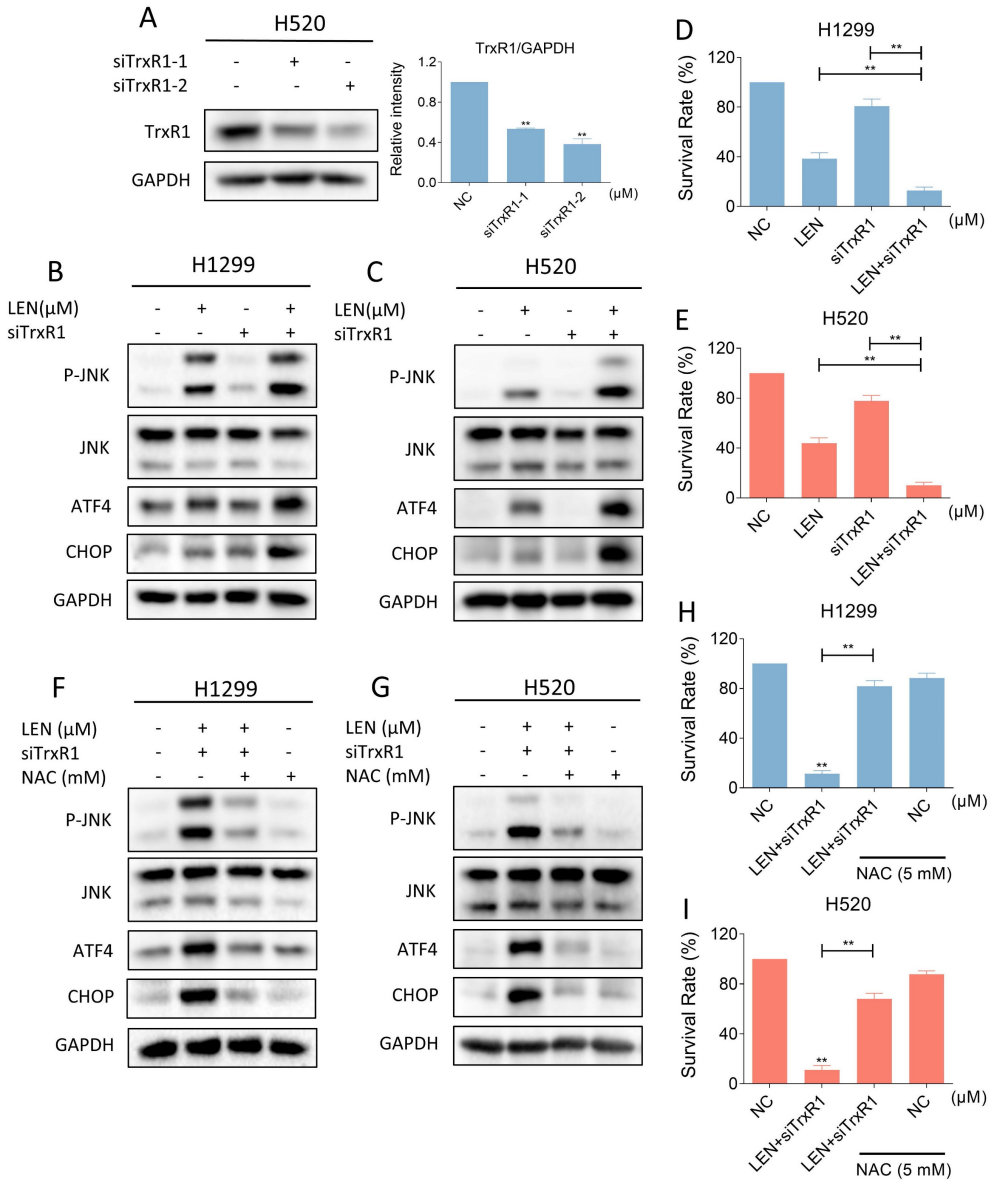
** Silencing of TrxR1 can synergize with lenvatinib to induce ROS mediated ER stress and JNK phosphorylation.** (A) The expression of TrxR1 in H520 cells with or without being treated by siTrxR1-1 or siTrxR1-2 was detected (** p < 0.01). (B-C) Western blot was used to analyze the expression of phosphorylated JNK, JNK, ATF4 and CHOP in H1299 and H520 cells after being treated by lenvatinib (40 μM) and siTrxR1 alone or the combination of them. (D-E) The viability of H1299 and H520 cells treated by lenvatinib (40 μM) and siTrxR1 alone or the combination of them was measured (** p < 0.01). (F-G) Cells with or without siTrxR1 were pre-treated by NAC for 1 hour and then treated by lenvatinib (40 μM), the level of p-JNK, JNK, ATF4 and CHOP in H1299, H520 or A549 cells was measured by western blot. (H-I) The H1299 and H520 cells with or without siTrxR1 were pre-treated by NAC for 1 hour and then treated by lenvatinib (40 μM), the viability was measured (** p < 0.01).

Considering the specificity of the TrxR1 inhibitor used in the study, we need to further confirm whether auranofin enhances the antitumor activity of LEN by inhibiting TrxR1 activity. We silenced TrxR1 in cells using siRNA (Figure 9A). Then we found that siTrxR1 and LEN can jointly upregulate the level of p-JNK, ATF4 and CHOP (Figure 9B-C and Figure S4A-B). The results of trypan blue counting showed that siTrxR1 and LEN synergistically inhibit lung cancer cell growth (Figure 9D-E). Moreover, pretreatment with NAC for 1 hour significantly counteracts the induction of p-JNK, ATF4 and CHOP expression caused by this combination, while also reversing cell death (Figure 9F-I and Figure S4C-D). These results proved that siTrxR1 can combine with LEN to cause endoplasmic reticulum stress and induce cell death, consistent with our previous data on the combination of TrxR1 inhibitors and LEN.

### LEN and AF synergistically inhibit tumor growth* in vivo*

**Figure 10:**
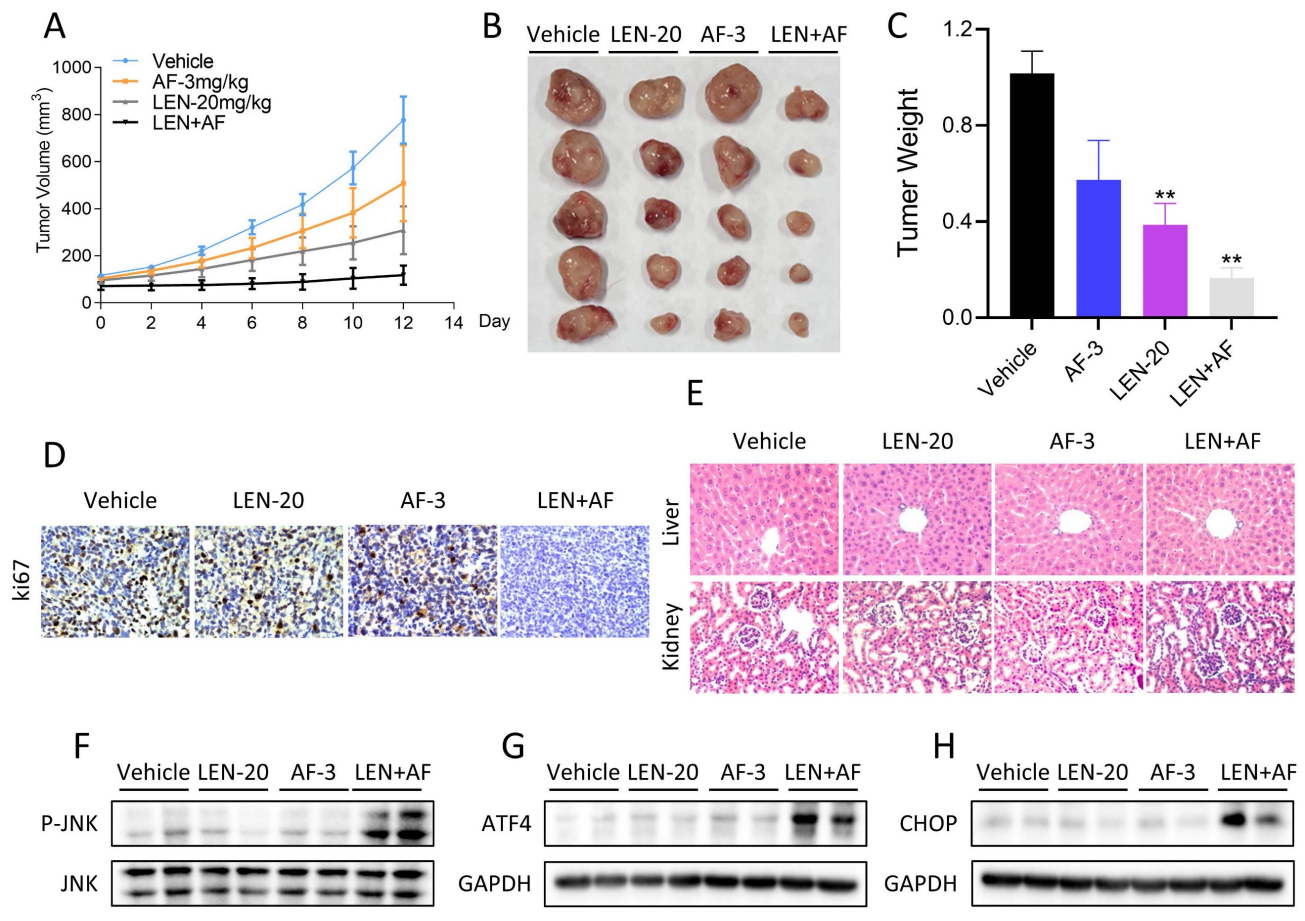
**Synergistic effects of lenvatinib and auranofin *in vivo*.** (A) The tumor volume of all nude mice was measured every two days starting from the beginning of administration. (B) Photos of xenograft tumors in each group. (C) Statistics of tumor weight in each group of mice (** p < 0.01). (D) IHC staining of Ki67 in tumors was conducted after being treated by lenvatinib (20 mg/kg) and auranofin (3 mg/kg) alone or the combination of them. (E) Histopathological analyses of organs were obtained by HE staining. (F) The level of p-JNK and JNK in tumors subjected to different treatments was detected by western blot. (G-H) The level of ATF4 and CHOP in tumors subjected to different treatments was detected by western blot.

Finally, we explored whether LEN and AF can synergistically inhibit tumor growth *in vivo*. The results indicated that the combination of LEN and AF can more significantly inhibit tumor growth *in vivo* compared to their individual use (Figure 10A-C). The result of immunohistochemistry also showed that LEN and AF can synergistically reduce the Ki67 level in tumors (Figure 10D). In order to confirm whether the combination has significant toxicity, we performed HE staining on the organ sections of nude mice. The histopathological analysis of organs revealed no significant toxicity in liver and kidney (Figure 10E). Subsequently, we conducted protein expression measurements in the tumors. Consistent with the expression pattern observed in cell experiments, LEN and AF synergistically activated the phosphorylation of JNK in tumors (Figure 10F). Besides, the expression of ATF4 and CHOP in tumors is also upregulated by combination treatment (Figure 10G-H).

## Discussion

Lung cancer has always been a major disease threatening human survival 36, 37. Lenvatinib, a multi-targeted tyrosine kinase inhibitor, has been approved by the FDA for treating liver cancer 38-40. The research of lenvatinib on KIF5B RET positive adenocarcinoma of the lung has entered phase II (nct01877083), and its clinical experiments combined with pembrolizumab on non-small cell lung cancer has entered phase III (nct03829332, nct04676412, nct03976375). However, lenvatinib is rarely used alone by reasons of its weak activity and drug resistance 41-43. Therefore, it is significant to find a combination regimen based on lenvatinib.

Previous studies have reported that TrxR1 inhibitors exert anti-cancer effects by increasing the level of ROS in cancer cells 27, 44. ROS is considered as a kind of regular metabolic by-product of cells, of which the content in tumors is usually higher than that in normal cells 45, 46. Therefore, tumor is more sensitive to the toxicity of ROS and it could be a good strategy to inhibit tumor by destroying the redox balance 47, 48. In this study, we found that TrxR1 inhibitors can significantly enhance the toxicity of VEGFR and EGFR inhibitors in lung cancer cells. After screening, we selected lenvatinib and auranofin for further research. 53BP1 is a protein involved in DNA repair, and its accumulation in the nucleus indicates DNA damage 49. We here observed that lenvatinib and auranofin can synergistically up-regulate ROS level and increase the nuclear 53BP1 foci in lung cancer cells. The pretreatment of NAC can eliminate the increase in ROS level and nuclear 53BP1 foci induced by lenvatinib combined with auranofin. These results confirm the conclusion that destroying the redox balance in tumor cells may be an efficacious anti-cancer strategy.

It has been shown that ROS accumulation can result in cell death by inducing ER stress 33, 34. Furthermore, ROS is a noteworthy activator of the JNK signaling pathway 30, 50. Not surprisingly, in the study, we found that lenvatinib and auranofin can synergistically induce ER stress and activate JNK signaling pathway. Besides, we found that JNK silencing as well as CHOP knockdown can partially reverse the cell death induced by lenvatinib combined with auranofin. This result indicates that the synergism between them is caused by the activation of JNK signaling pathway and ER stress, which is mediated by the accumulation of ROS.

Considering the limitations of TrxR1 inhibitors, we used siTrxR1 to verify the previous conclusions. The results showed that TrxR1 silencing can significantly enhance the antitumor activity of lenvatinib and synergistically induce ER stress and JNK phosphorylation. These findings are consistent with previous results that demonstrated a synergistic effect between TrxR1 inhibitors and lenvatinib. It has been reported that lenvatinib can induce ROS generation by inhibiting FGFR4 51. Moreover, TrxR1 inhibitors can inhibit the activity of TrxR1 in cells and weaken the ability of cells to scavenge ROS and maintain redox homeostasis 52. Therefore, we speculate that TrxR1 inhibitors can further upregulate the ROS level induced by lenvatinib. However, whether the synergistic effect between TrxR1 inhibitor and lenvatinib is related to the RTK inhibitory effect of lenvatinib, and the synergistic effect between TrxR1 inhibitor and other RTK inhibitors need to be further studied.

## Conclusions

**Figure 11:**
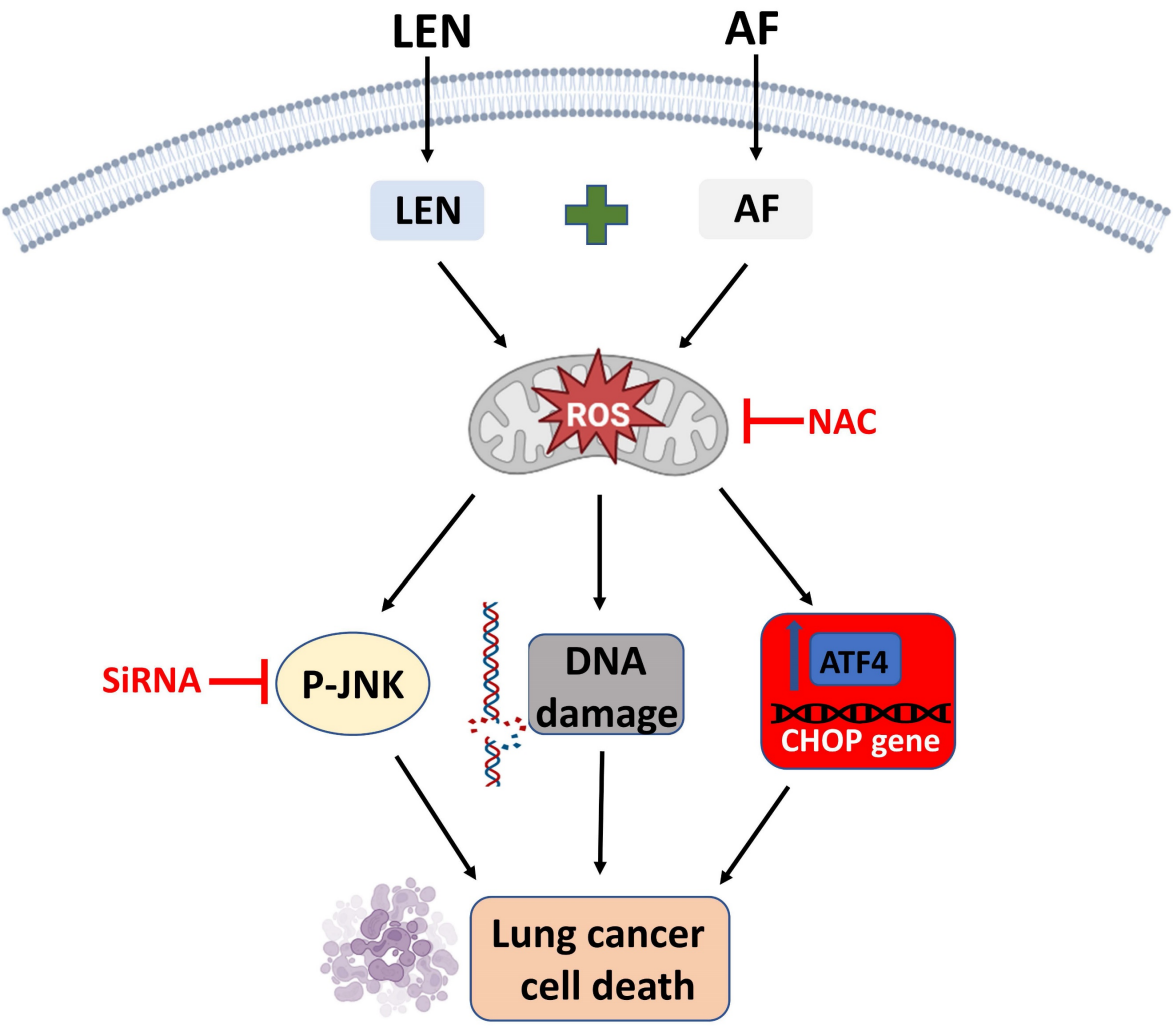
The proposed working model.

To sum up, we have proved that lenvatinib and auranofin can cause DNA damage and eventually cell death by synergistically up-regulating ROS level in lung cancer cells (Figure 11). Our findings serve a novel drug combination for the clinical treatment of lung cancer. The discovery that TrxR1 inhibitors enhance the sensitivity of lung cancer cells to VEGFR inhibitor provides a new idea for clinical chemotherapy drug regimen.

## Supplementary Material

Supplementary figures.

## Abbreviations

VEGFR: Vascular endothelial growth factor receptor
TrxR1: Thioredoxin reductase 1
LEN: Lenvatinib
AF: Auranofin
CED: Cediranib
ELO: Erlotinib
NIN: Nintedanib
PL: Piperlongumine
ROS: Reactive oxygen species
FBS: Fetal bovine serum
NAC: N-acetyl-L-cysteine
ECL: Enhanced chemiluminescence
siRNA: Small interfering RNA
