# Supplementary material for: Combination of TrxR1 inhibitor and lenvatinib triggers ROS-dependent cell death in human lung cancer cells
Source: Int J Biol Sci. 2024 Jan 1;20(1):249–64. doi: 10.7150/ijbs.86160 (PMC10750290; doi:10.7150/ijbs.86160)
Supplement: Supplementary file 1 — Supplementary figures. [file ijbsv20p0249s1.pdf]

Fig.S1

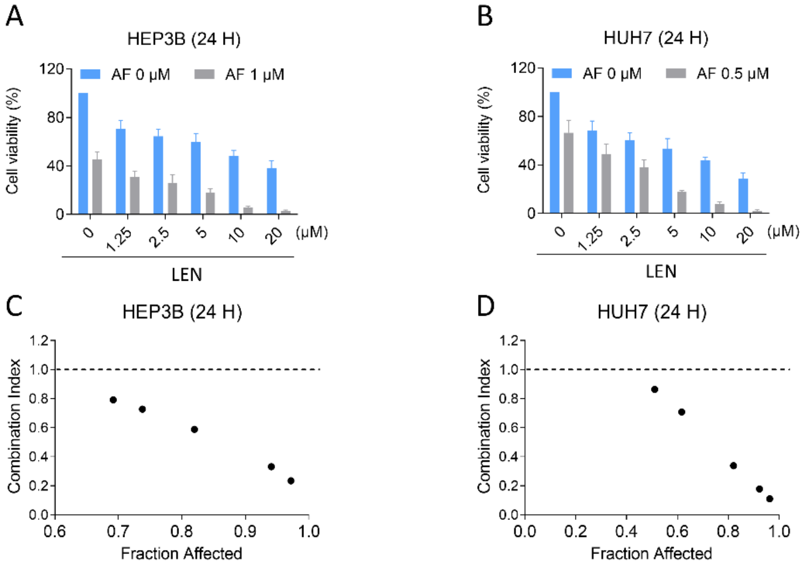

Supplementary Fig 1. Lenvatinib and auranofin synergistically induce hepatocellular carcinoma cell death. (A-B) Viability of HEP3B and HUH7 cells treated by different concentrations of lenvatinib with/without auranofin were measured. (C-D) CompuSyn software was used to calculate CI values.

Fig.S2

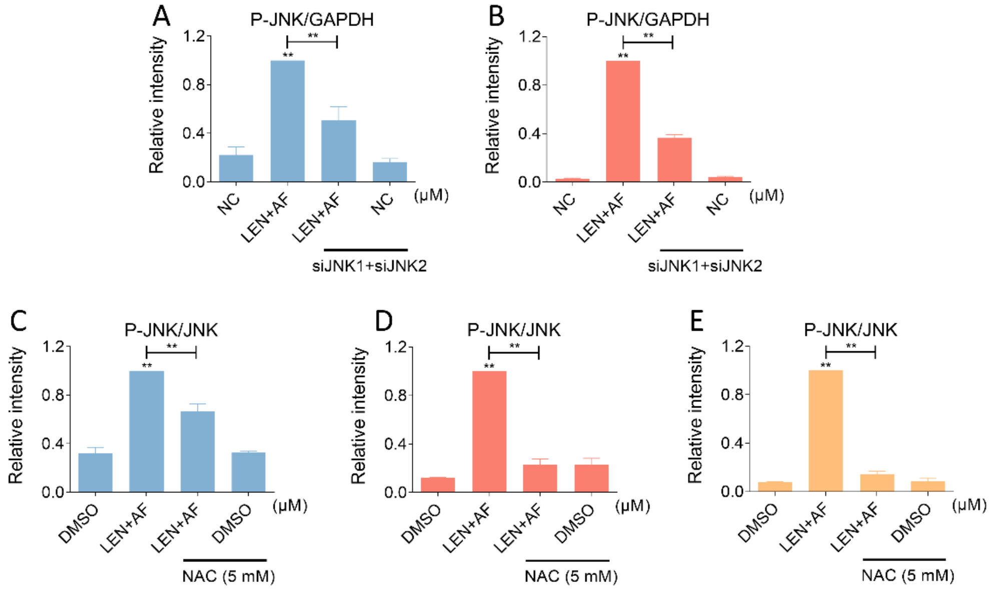

Supplementary Fig 2. NAC and siJNK can reverse the phosphorylation of JNK. (A-B) The expression of phosphorylated JNK and JNK in H1299 (LEN 10 μM, AF 0.8 μM) and H520 (LEN 10 μM, AF 1.5 μM) cells were detected after being treated by the combination of lenvatinib and auranofin with or without siJNK1 and siJNK2 (** $p < 0.01$). (C-E) Cells were treated by NAC for 1 hour before the combination treatment, western blot was used to analyze the expression of phosphorylated JNK and JNK in

H1299 (LEN 10 µM, AF 0.8 µM), H520 (LEN 10 µM, AF 1.5 µM) or A549 (LEN 20 µM, AF 4 µM) cells (** $p < 0.01$).

Fig.S3

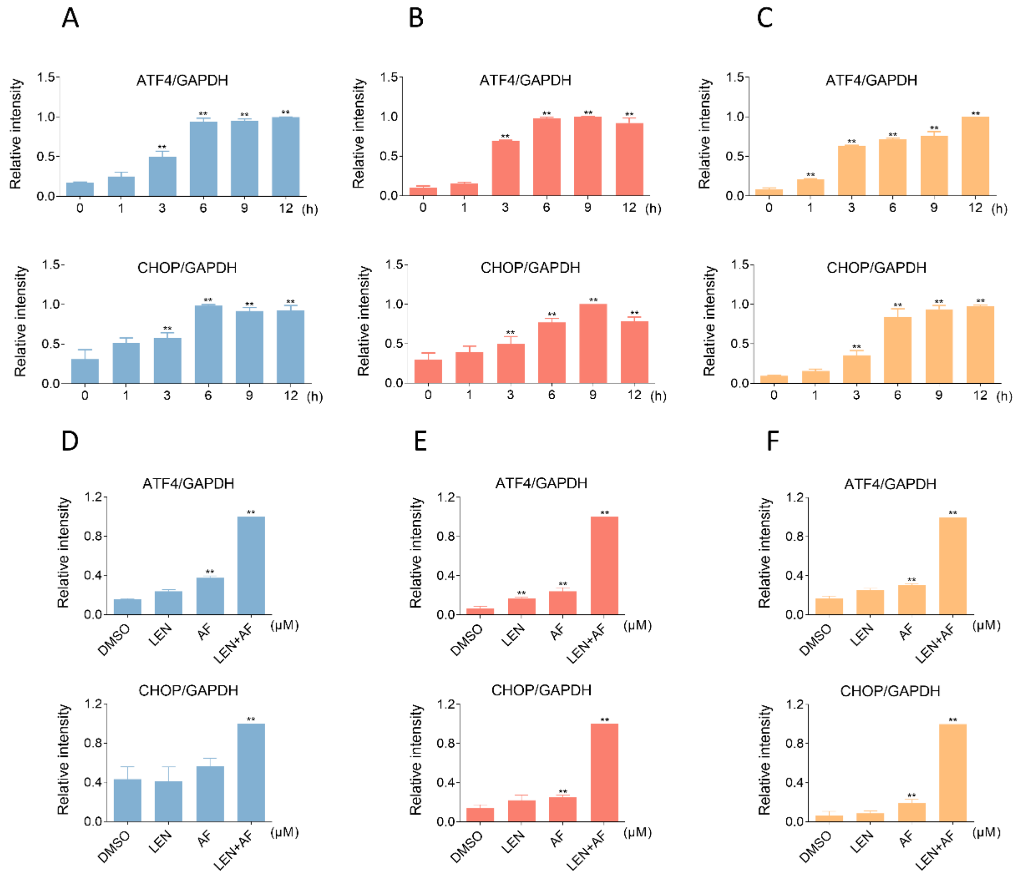

Supplementary Fig 3. The combination of lenvatinib and auranofin can lead to ER stress. (A-C) The expression of ATF4 and CHOP in H1299 (LEN 10 µM, AF 0.8 µM), H520 (LEN 10 µM, AF 1.5 µM) and A549 (LEN 20 µM, AF 4 µM) cells was detected after being treated by the combination of lenvatinib and auranofin for different durations (** $p < 0.01$). (D-F) Western blot was used to analysis the expression of ATF4 and CHOP in H1299 (LEN 10 µM, AF 0.8 µM), H520 (LEN 10 µM, AF 1.5 µM) or A549 (LEN 20 µM, AF 4 µM) cells after being treated by lenvatinib and auranofin alone or the combination of them (** $p < 0.01$).

Fig.S4

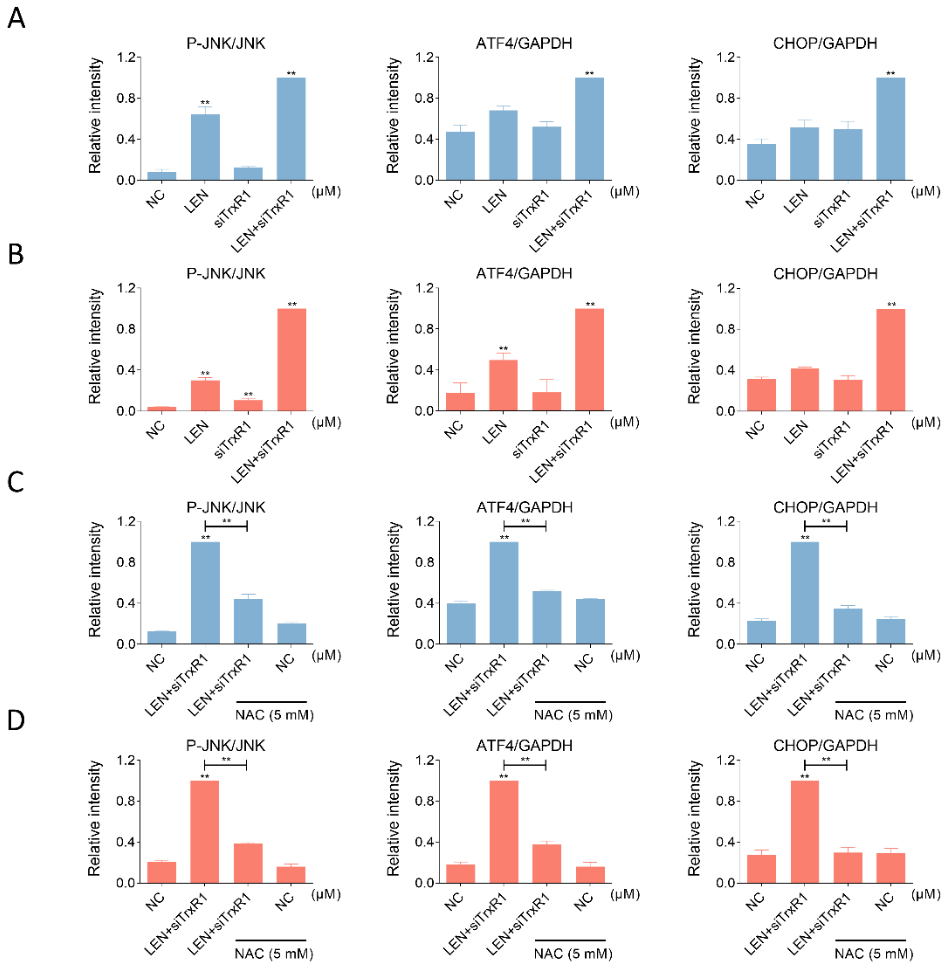

Supplementary Fig 4. Silencing of TrxR1 and lenvatinib can jointly induce ROS-dependent ER stress and JNK phosphorylation. (A-B) Western blot was used to analysis the expression of phosphorylated JNK, JNK, ATF4 and CHOP in H1299 and H520 cells after being treated by lenvatinib (LEN 40 μM) and siTrxR1 alone or the combination of them (** $p < 0.01$). (C-D) Cells with or without siTrxR1 were pretreated by NAC for 1 h and then treated by lenvatinib (LEN 40 μM), the expression of phosphorylated JNK, JNK, ATF4 and CHOP in H1299, H520 or A549 cells were detected by western blot (** $p < 0.01$).
